# 

**Published:** 1908-04-18

**Authors:** 


### CONTENTS.

| | PAGE |
|---|---|
| **The Voluntary Hospitals, Patients, Payments and State Aid** ... | 55 |
| **The Sense of Death** ... | 56 |
| **Annotations—** The Gyroscope at Sea—A "Doctor's Dilemma"—Iron and the Teeth—Medical Superintendents' Salaries ... | 57 |
| **Medical Opinion and Movement** ... | 58 |
| **Hospital Clinics—** Hyperemesis Gravidarum. By Sir J. Halliday Croom, M.D., F.R.C.P., F.R.C.S. ... | 61 |
| **Special Article—** Perineal Prostatectomy ... | 63 |
| **Medicine—** Acute Dilatation of the Stomach ... | 65 |
| **Points in Treatment—** The Vaccine Treatment of Gonococcal Arthritis ... | 66 |
| Mercurial Cream for Injection in Syphilis... | 66 |
| **Points in Surgery—** Some Aspects of General Peritonitis—II. ... | 67 |
| **Diseases of Children—** The Apex Beat of the Heart ... | 68 |
| **Mental Diseases—** Two Cases of Restlessness—II. ... | 69 |
| **Therapeutics—** Secret Remedies and Branded Products—V. ... | 70 |
| **"The Hospital" Medical Book Supplement.—No. VI.** ... | 71 |
| **Hospital Administration—** Fiji: its Hospitals and Diseases—II. ... | 75 |
| News and Coming Events ... | 77 |
| **Nursing Administration—** The Common Task ... | 79 |
| **Editor's Letter-Box—** A Uniform Certificate for Hospital Nurses ... | 80 |
| **New Appliances and Things Medical—** Some Alkaloidal Specialities ... | 80 |

